# Preservation of kidney function irrelevant of total kidney volume growth rate with tolvaptan treatment in patients with autosomal dominant polycystic kidney disease

**DOI:** 10.1007/s10157-020-02009-0

**Published:** 2021-01-20

**Authors:** Shigeo Horie, Satoru Muto, Haruna Kawano, Tadashi Okada, Yoshiyuki Shibasaki, Koji Nakajima, Tatsuki Ibuki

**Affiliations:** 1 Department of Urology, Juntendo University Graduate School of Medicine, 2-1-1 Hongo, Bunkyo-ku, Tokyo, 113-8421 Japan; 2 Department of Advanced Informatics for Genetic Diseases, Juntendo University Graduate School of Medicine, Tokyo, Japan; 3 Department of Clinical Development, Otsuka Pharmaceutical Co., Ltd, Osaka, Japan; 4 Medical Affairs, Otsuka Pharmaceutical Co., Ltd, Tokyo, Japan

**Keywords:** Autosomal dominant polycystic kidney disease, Glomerular filtration rate, Japan, Tolvaptan, Total kidney volume

## Abstract

**Background:**

Tolvaptan slowed the rates of total kidney volume (TKV) growth and renal function decline over a 3-year period in patients with autosomal dominant polycystic kidney disease (ADPKD) enrolled in the Tolvaptan Efficacy and Safety in Management of Autosomal Dominant Polycystic Kidney Disease and Its Outcomes (TEMPO) 3:4 trial (NCT00428948). In this post hoc analysis of Japanese patients from TEMPO 3:4, we evaluated whether the effects of tolvaptan on TKV and on renal function are interrelated.

**Methods:**

One hundred and forty-seven Japanese patients from TEMPO 3:4 were included in this analysis (placebo, *n* = 55; tolvaptan, *n* = 92). Tolvaptan-treated patients were stratified into the responder group (*n* = 37), defined as tolvaptan-treated patients with a net decrease in TKV from baseline to year 3, and the non-responder group (*n* = 55), defined as tolvaptan-treated patients with a net increase in TKV.

**Results:**

Mean changes during follow-up in the placebo, responder, and non-responder groups were 16.99%, − 8.33%, and 13.95%, respectively, for TKV and − 12.61, − 8.47, and − 8.58 mL/min/1.73 m^2^, respectively, for estimated glomerular filtration rate (eGFR). Compared with the placebo group, eGFR decline was significantly slowed in both the responder and non-responder groups (*P* < 0.05).

**Conclusion:**

Tolvaptan was effective in slowing eGFR decline, regardless of TKV response, over 3 years in patients with ADPKD in Japan. Treatment with tolvaptan may have beneficial effects on slowing of renal function decline even in patients who have not experienced a reduction in the rate of TKV growth by treatment with tolvaptan.

**Supplementary Information:**

The online version contains supplementary material available at 10.1007/s10157-020-02009-0.

## Introduction

Autosomal dominant polycystic kidney disease (ADPKD) is an inherited disease characterized by age-dependent development of multiple cysts in the kidneys, causing a gradual and irreversible expansion of kidney volume [1]. ADPKD eventually leads to end-stage renal failure and the need for renal replacement therapy in a majority of patients, typically by the fifth or sixth decade of life [2]. Natural history data on the gradual expansion of total kidney volume (TKV) in ADPKD are available, including 8 years of follow-up from the prospective, observational Consortium for Radiologic Imaging Studies of Polycystic Kidney Disease (CRISP) I and II studies [1, 3, 4]. TKV and glomerular filtration rate (GFR) are markers of ADPKD progression, with TKV used as a prognostic indicator early in the disease course, prior to significant changes in renal function [4]. Tolvaptan, a vasopressin V_2_-receptor antagonist, suppresses binding of vasopressin to the V_2_-receptor in renal epithelial cells, and vasopressin V_2_-receptor blockade has been shown, in animal models of ADPKD, to inhibit the growth of renal cysts and the decline in renal function [5–9]. In the Tolvaptan Efficacy and Safety in Management of Autosomal Dominant Polycystic Kidney Disease and Its Outcomes (TEMPO) 3:4 clinical trial, a global, multicenter study that included patients from Japan, tolvaptan slowed the rates of TKV growth and renal function decline over a 3-year period compared with placebo in patients with ADPKD [10, 11].

In this post hoc analysis of patients enrolled in TEMPO 3:4 in Japan, we assessed the patterns of TKV response to tolvaptan in ADPKD and explored the factors potentially predictive of TKV response, such as baseline ADPKD risk class [12]. Further, we evaluated possible correlations between the effects of tolvaptan on TKV growth rate and on renal function decline.

## Materials and methods

### Study design

This study is a post hoc analysis of the Japanese population from TEMPO 3:4, a multicenter, double-blind, placebo-controlled, 3-year clinical study (ClinicalTrials.gov number, NCT00428948) [10, 11]. Patients were enrolled at 129 sites worldwide. Study design, setting, data collection, and enrollment criteria have been previously described in detail [10, 13]. Briefly, eligible patients were aged 18–50 years, with a diagnosis of ADPKD by the Ravine criteria, TKV ≥ 750 mL as measured by magnetic resonance imaging (MRI), and estimated creatinine clearance (Ccr) ≥ 60 mL/min. Patients were randomly assigned in a 2:1 ratio to receive tolvaptan or placebo. Study drug was titrated from a daily split dose of 45/15 mg–60/30 mg and 90/30 mg based on patient-reported tolerability. After the titration phase, patients took the highest dose tolerable for 36 months. The primary endpoint was the annual rate of change in TKV from baseline; secondary endpoints included a composite of time to clinical progression (defined as worsening kidney function, kidney pain, hypertension, and albuminuria) and rate of kidney function decline.

TEMPO 3:4 was conducted in accordance with the ethical principles originating in the Declaration of Helsinki and in compliance with good clinical practice guidelines. The protocol was approved by the institutional review board at each trial site. Written informed consent was obtained from all participants.

### Objectives

The objectives of this post hoc analysis were to compare the change in TKV from baseline to year 3 among the placebo, responder, and non-responder groups; identify the factors predictive of decrease in TKV; and explore the correlations between changes in TKV and kidney function.

### Patients

Patients who completed the TEMPO 3:4 trial in Japan were stratified into three groups: (1) placebo-treated patients; (2) responders, defined as tolvaptan-treated patients with a net decrease in TKV from baseline to year 3; and (3) non-responders, defined as tolvaptan-treated patients who showed a net increase in TKV.

### Evaluations

Data from the end of the tolvaptan titration phase were used for baseline kidney function values. Ccr was calculated by the Cockcroft–Gault formula [14]. Calculation of estimated glomerular filtration rate (eGFR; mL/min/1.73 m^2^) was performed as follows by two formulae: eGFR by the Chronic Kidney Disease Epidemiology Collaboration (CKD-EPI) equation modified for Japanese = 0.813 (Japanese coefficient) × 141 × minimum (serum creatinine/κ or 1)^α^ × maximum (serum creatinine/κ or 1)^−1.209^ × 0.993^Age^ × 1.018 (if female) × 1.159 (if black) (κ is 0.7 for females and 0.9 for males, α is − 0.329 for females and − 0.411 for males) [15] and eGFR by the Japanese eGFR equation based on serum creatinine (eGFR-J) developed by the Japanese Society of Nephrology = 194 × serum creatinine^−1.094^ × age^−0.287^ (if male) and 194 × serum creatinine^−1.094 ^× age^−0.287^ × 0.739 (if female) [16]. Chronic kidney disease stage was classified based on GFR [17].

Baseline MRI data were assessed in blinded reads to categorize patients into ADPKD risk classes according to prespecified imaging criteria [12]. Patients were categorized as class 1 (typical ADPKD) or class 2 (atypical ADPKD), with class 1 patients further stratified into five subclasses (1A–1E) based on height-adjusted TKV (htTKV) and age. The estimated annual kidney growth rates for each subclass are < 1.5% (1A), 1.5–3.0% (1B), 3.0–4.5% (1C), 4.5–6.0% (1D), and > 6.0% (1E), with a theoretical initial htTKV of 150 mL/m. In the analysis of predictive factors for change in TKV, ADPKD risk class was expressed on an ordinal scale from class 1B to 1E.

### Statistical analysis

The proportions of patients with a net decrease in TKV from baseline to year 3 in the placebo and tolvaptan groups were compared by the chi-square test. Factors affecting decrease in TKV were analyzed by univariate logistic regression, with calculation of odds ratio (OR), 95% confidence interval (CI), and *P* values. The covariates analyzed included demographic characteristics, stratification factors, medical history, current medication, polycystic kidney disease characteristics, and kidney function parameters. Factors found to be significant were further analyzed in a multiple logistic regression model.

To evaluate the correlations between changes in TKV and changes in kidney function parameters from baseline to year 3, Pearson’s correlation coefficient (*r*), simple regression analysis parameter estimate, standard error, 95% CI, and *P* value were calculated. Changes in TKV, kidney function, and urine osmolality from baseline to year 3 were compared among the placebo and tolvaptan groups using Tukey–Kramer’s honestly significant difference test.

Statistical significance was defined as *P* < 0.05. All statistical analyses were performed using SAS 9.4 and JMP 13 (SAS Institute, Cary, North Carolina).

## Results

### Patient characteristics

**Fig. 1:**
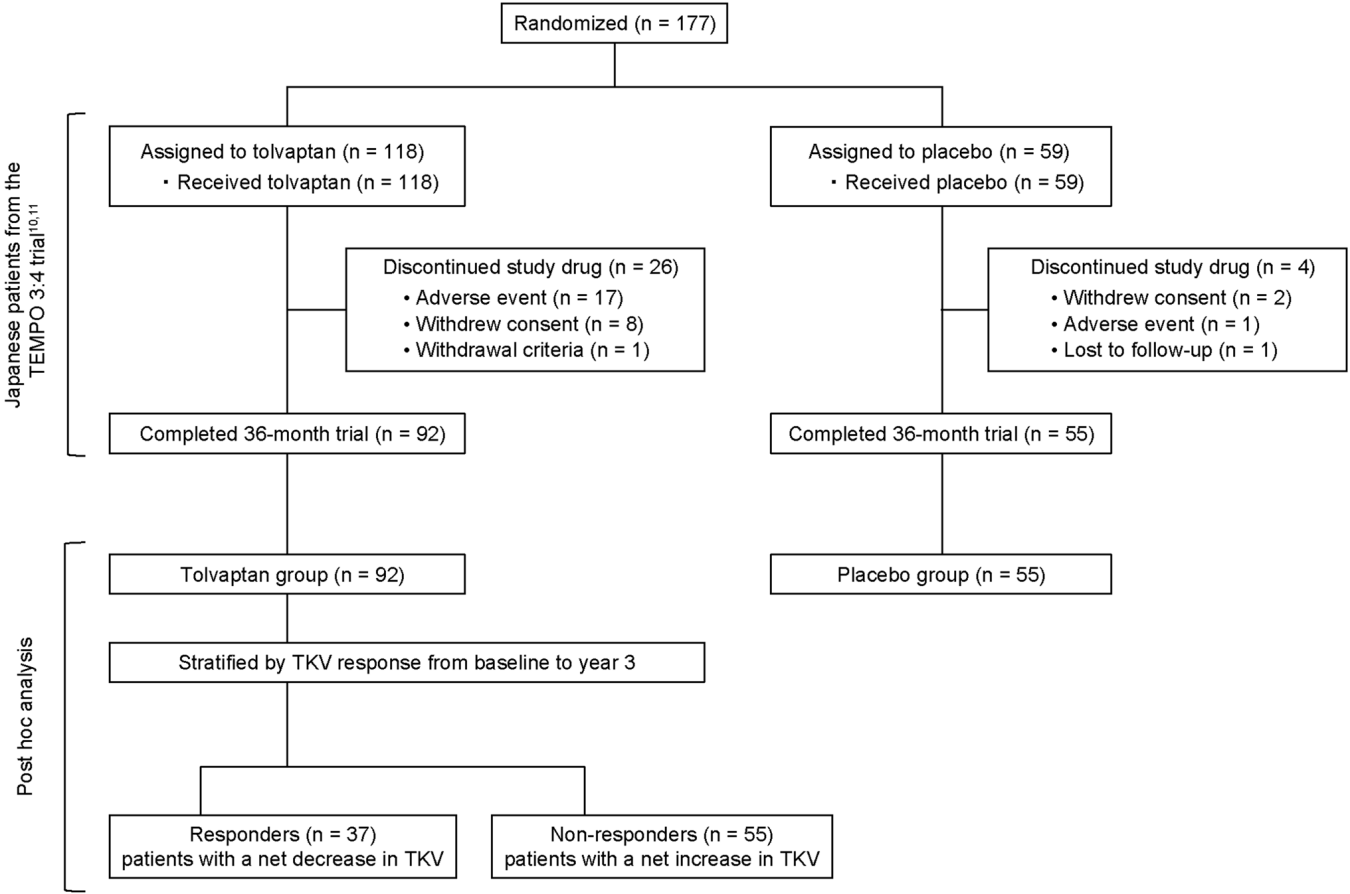
Patient flow and analysis set. Patients who received tolvaptan in the TEMPO 3:4 trial in Japan were stratified into two groups. *TKV* total kidney volume

**Fig. 2:**
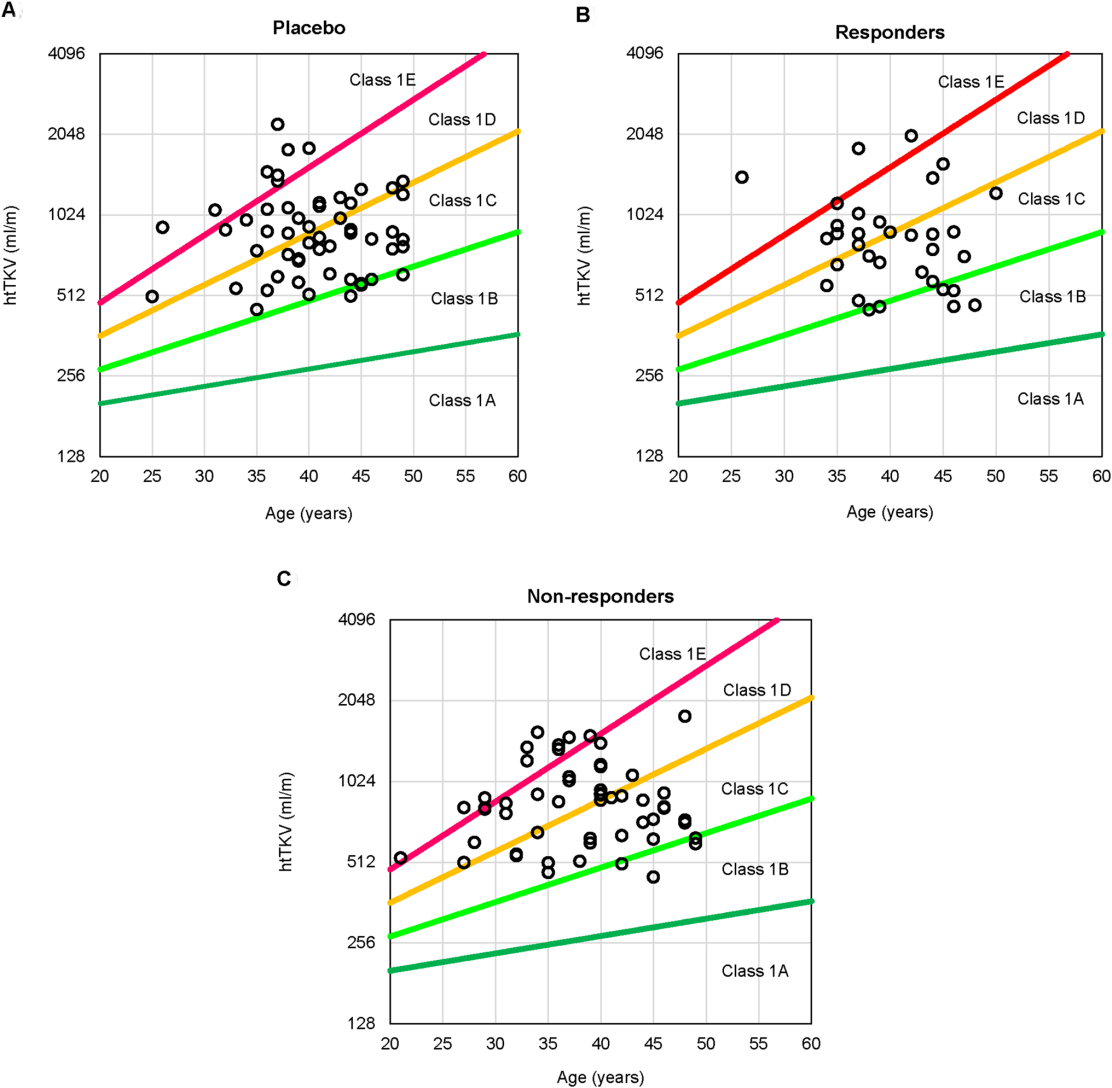
Risk classification by htTKV and age in class 1 patients. Vertical scale is a log_2_ scale. Plots are baseline values of htTKV and age. Class 1 was stratified into five subclasses (1A–1E) based on estimated kidney growth rates: yearly rates of increase of < 1.5% (1A), 1.5–3.0% (1B), 3.0–4.5% (1C), 4.5–6.0% (1D), and > 6.0% (1E), with a theoretical initial htTKV of 150 mL/m. Responders: tolvaptan-treated patients with a net decrease in TKV from baseline to year 3. Non-responders: tolvaptan-treated patients with a net increase in TKV from baseline to year 3. *htTKV* height-adjusted total kidney volume, *TKV* total kidney volume

One hundred and forty-seven Japanese patients from the TEMPO 3:4 trial were included in this analysis (placebo, *n* = 55; tolvaptan, *n* = 92). By ADPKD risk classification, 9.5% of patients were class 1B, 40.8% class 1C, 30.6% class 1D, and 16.3% class 1E. Tolvaptan-treated patients were stratified into responders (*n* = 37), defined as patients who achieved a net decrease in TKV from baseline to year 3, or non-responders (*n* = 55), defined as patients who experienced a net increase in TKV from baseline to year 3 (Fig. 1). Patient characteristics and demographic data in the placebo, responder, and non-responder groups are shown in Table 1. The distribution of patients in each risk subclass was similar between the placebo and tolvaptan groups (Fig. 2). Overall, 40.2% (37/92) of tolvaptan-treated patients, compared with 5.5% (3/55) of placebo-treated patients, experienced a net decrease in TKV from baseline to year 3 (*P* < 0.0001). Tolvaptan was associated with decreased TKV in 60% of class 1B patients (6 responders among 10 tolvaptan-treated patients), 38.2% of class 1C patients (13 responders among 34 tolvaptan-treated patients), 39.3% of class 1D patients (11 responders among 28 tolvaptan-treated patients), and 18.8% of class 1E patients (3 responders among 16 tolvaptan-treated patients). Calcium channel blockers were prescribed in significantly fewer patients in the responder group than in either the placebo (*P* = 0.0216) or the non-responder (*P* = 0.0490) group.

**Table 1:** Patient characteristics and demographic data

	Placebo (*n* = 55)	Tolvaptan (*n* = 92)	
	Placebo group	Responders group	Non-responders group
	(*n* = 55)	(*n* = 37)	(*n* = 55)
Demographic characteristic			
Sex (male)	33 (60.0)	12 (32.4)	36 (65.5)
Age (years)	40.5 ± 5.6	39.7 ± 5.4	38.1 ± 6.5
Height (cm)	169.5 ± 6.9	164.0 ± 8.8	169.3 ± 8.2
Weight (kg)	66.4 ± 13.1	61.2 ± 12.5	69.2 ± 12.2
BMI (kg/m^2^)	23.0 ± 3.4	22.6 ± 2.9	24.0 ± 3.3
Polycystic kidney disease characteristic			
Systolic blood pressure (mm Hg)	125.4 ± 12.8	123.2 ± 12.2	127.4 ± 14.6
Diastolic blood pressure (mm Hg)	80.8 ± 9.8	79.5 ± 11.3	81.9 ± 14.1
Mean blood pressure (mm Hg)	95.7 ± 10.0	94.1 ± 11.0	97.0 ± 13.2
TKV (mL)	1582.2 ± 643.8	1434.3 ± 640.6	1518.9 ± 559.8
Height-adjusted TKV (mL/m)	928.5 ± 362.1	871.7 ± 377.9	897.7 ± 327.3
Cr (mg/dL)	0.97 ± 0.27	0.95 ± 0.32	1.03 ± 0.32
1/Cr [(mg/mL)^−1^]	111.7 ± 33.7	115.7 ± 34.4	106.4 ± 32.4
Ccr (mL/min)^a^	93.1 ± 23.1	84.3 ± 21.8	95.1 ± 27.0
eGFR CKD-EPI (mL/min/1.73 m^2^)^b^	73.7 ± 16.4	71.3 ± 17.2	72.0 ± 17.7
eGFR-J (mL/min/1.73 m^2^)^c^	66.3 ± 17.1	63.6 ± 16.5	65.0 ± 17.0
Urine albumin-to-creatinine ratio	10.7 ± 18.3	7.5 ± 7.8	11.2 ± 16.5
CysC (mg/L)	0.77 ± 0.19	0.76 ± 0.20	0.80 ± 0.20
Urine osmolality (mOsm/kg)	475.0 ± 137.1	440.8 ± 140.6	465.7 ± 151.2
CKD stage^d^			
G1	3 (5.5)	2 (5.4)	5 (9.3)
G2	31 (56.4)	20 (54.1)	25 (46.3)
G3a	17 (30.9)	8 (21.6)	18 (33.3)
G3b	4 (7.3)	7 (18.9)	6 (11.1)
G4	0 (0.0)	0 (0.0)	0 (0.0)
G5	0 (0.0)	0 (0.0)	0 (0.0)
ADPKD risk classification^e^			
1A	0 (0.0)	0 (0.0)	0 (0.0)
1B	4 (7.3)	6 (16.2)	4 (7.3)
1C	26 (47.3)	13 (35.1)	21 (38.2)
1D	17 (30.9)	11 (29.7)	17 (30.9)
1E	8 (14.5)	3 (8.1)	13 (23.6)
2A/2B	0 (0.0)	4 (10.8)	0 (0.0)
Medical history			
Hematuria	16 (29.1)	7 (18.9)	12 (21.8)
Kidney pain	4 (7.3)	4 (10.8)	6 (10.9)
Nephrolithiasis	7 (12.7)	2 (5.4)	6 (10.9)
Urinary tract infection	3 (5.5)	4 (10.8)	4 (7.3)
Anemia	3 (5.5)	4 (10.8)	5 (9.1)
Proteinuria	20 (36.4)	12 (32.4)	14 (25.5)
Current medication			
ACE inhibitor	8 (14.5)	2 (5.4)	3 (5.5)
ARB	36 (65.5)	17 (45.9)	36 (65.5)
ACE inhibitor, ARB, or both	38 (69.1)	18 (48.6)	37 (67.3)
Beta-blocker	7 (12.7)	2 (5.4)	7 (12.7)
Calcium channel blocker	23 (41.8)	7 (18.9)	21 (38.2)
Diuretic	2 (3.6)	0 (0.0)	2 (3.6)

Categorical data are expressed as number (%). Continuous data are expressed as mean ± standard deviation. Responders: tolvaptan-treated patients with a net decrease in TKV from baseline to year 3. Non-responders: tolvaptan-treated patients with a net increase in TKV from baseline to year 3

*1/Cr* reciprocal of serum creatinine, *ACE* angiotensin-converting enzyme, *ADPKD* autosomal dominant polycystic kidney disease, *ARB* angiotensin II receptor blocker, *BMI* body mass index, *Ccr* estimated creatinine clearance, *CKD* chronic kidney disease, *CKD-EPI* Chronic Kidney Disease Epidemiology Collaboration, *Cr* serum creatinine, *CysC* serum cystatin C, *eGFR* estimated glomerular filtration rate, *eGFR-J* estimated glomerular filtration rate by the Japanese equation based on serum creatinine, *TKV* total kidney volume

^a^Ccr calculated by the Cockcroft–Gault formula

^b^eGFR calculated by the CKD-EPI equation modified for Japanese

^c^eGFR calculated by the Japanese eGFR equation based on Cr, as developed by the Japanese Society of Nephrology

^d^CKD stage was classified based on GFR category (in mL/min/1.73 m^2^): G1, ≥ 90; G2, 60–89; G3a, 45–59; G3b, 30–44; G4, 15–29; G5, < 15

^e^Patients were categorized into class 1 (typical ADPKD) or class 2 (atypical ADPKD) based on baseline imaging findings, and class 1 was stratified into five subclasses (1A–1E) based on height-adjusted TKV and age

### Change in TKV

**Fig. 3:**
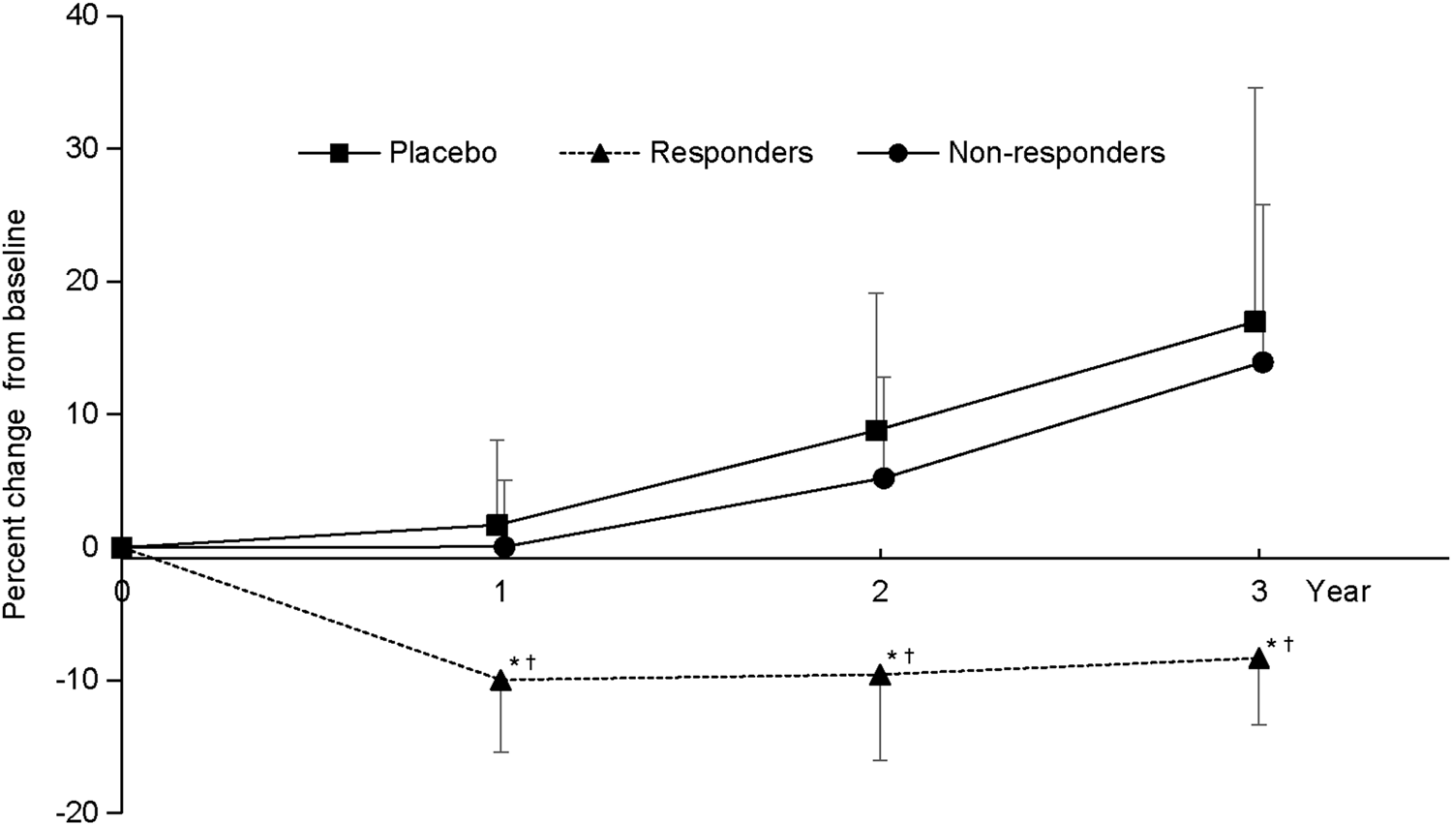
Time course of change in TKV over 3 years in the placebo, responder, and non-responder groups. Values are expressed as mean and standard deviation. Responders: tolvaptan-treated patients with a net decrease in TKV from baseline to year 3. Non-responders: tolvaptan-treated patients with a net increase in TKV from baseline to year 3. *P* < 0.0001 based on Tukey–Kramer’s honestly significant difference test comparing percent change in TKV from baseline to each year of follow-up between the placebo and responder groups (*) and between the responder and non-responder groups (^†^). *TKV* total kidney volume

**Fig. 4:**
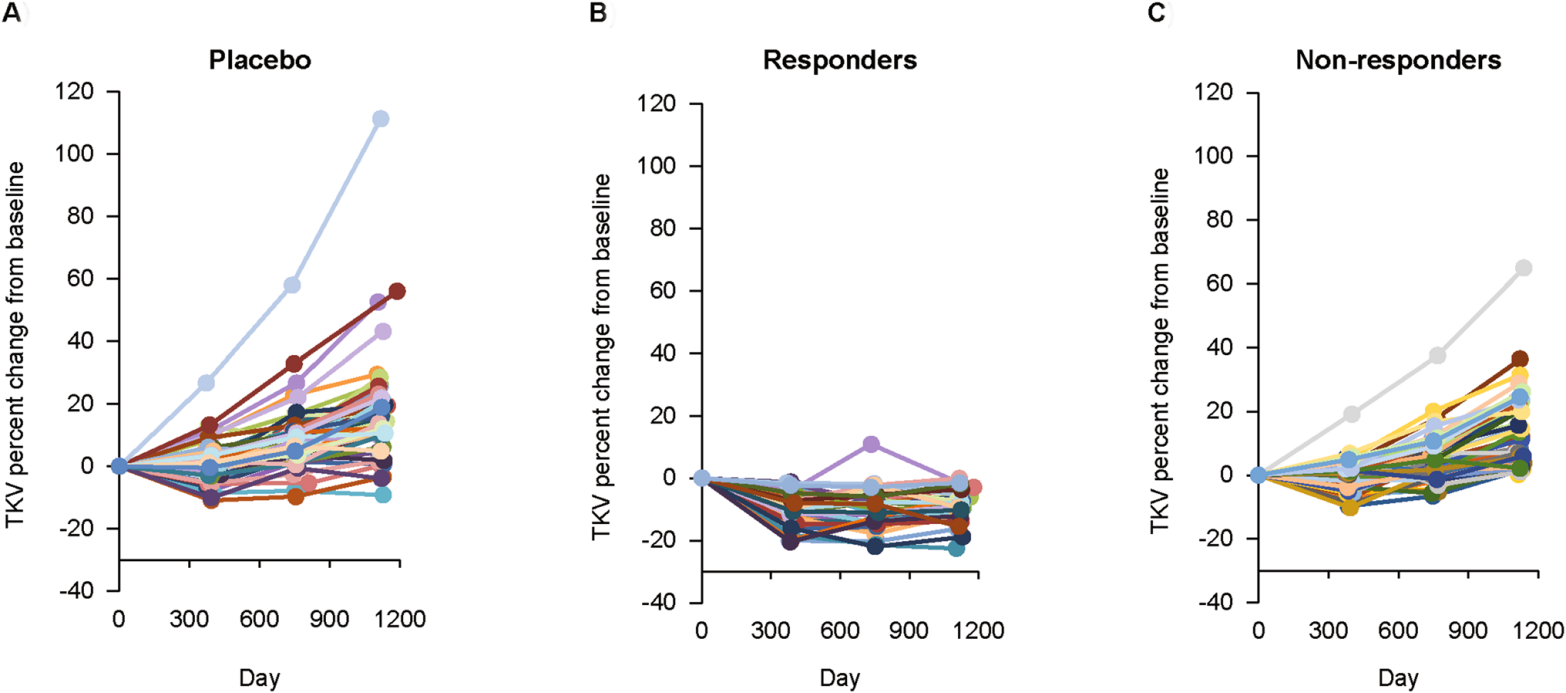
Time course of TKV in each patient throughout the treatment period. Responders: tolvaptan-treated patients with a net decrease in TKV from baseline to year 3. Non-responders: tolvaptan-treated patients with a net increase in TKV from baseline to year 3. *TKV* total kidney volume

The time course of percent change in TKV at each visit is shown in Fig. 3 for each group. Changes in TKV from baseline to each year of follow-up were significantly different between the placebo and responder groups (*P* < 0.0001) and between the responder and non-responder groups (*P* < 0.0001). No statistically significant differences between the placebo and non-responder groups were observed. In the responder group, TKV was decreased in all patients at 12 months, with the decreases maintained throughout 3 years of treatment in all patients, with the exception of one patient at year 2 (Fig. 4).

### Predictors of TKV decrease

The significant predictive factors for TKV decrease with tolvaptan were sex (OR for males 0.25; 95% CI 0.10–0.61; *P* = 0.0024), height (OR 0.93; 95% CI 0.88–0.98; *P* = 0.0063), weight (OR 0.95; 95% CI 0.91–0.98; *P* = 0.0050), and body mass index (BMI; OR 0.85; 95% CI 0.73–0.99; *P* = 0.0333) (Table 2). Multiple logistic regression analysis was performed using two factors, sex and BMI. Sex was a significant predictor of TKV response (OR for male sex 0.31; 95% CI 0.12–0.81; *P* = 0.0165) (Table 3).

**Table 2:** Univariate analysis of factors predictive of TKV reduction

	Odds ratio	95% CI	*P* value
Demographic characteristic			
Sex (male)	0.25	0.10–0.61	0.0024
Age (years)	1.05	0.97–1.12	0.2093
Height (cm)	0.93	0.88–0.98	0.0063
Weight (kg)	0.95	0.91–0.98	0.0050
BMI (kg/m^2^)	0.85	0.73–0.99	0.0333
Stratification factor			
Hypertension	0.51	0.20–1.26	0.1453
Ccr < 80 mL/min	2.36	1.00–5.59	0.0509
TKV < 1500 mL	1.69	0.64–4.45	0.2859
Medical history			
Hematuria	0.84	0.29–2.37	0.7364
Kidney pain	0.99	0.26–3.78	0.9882
Nephrolithiasis	0.47	0.09–2.45	0.3676
Urinary tract infection	1.55	0.36–6.61	0.5572
Anemia	1.21	0.30–4.85	0.7856
Proteinuria	1.41	0.56–3.52	0.4670
Current medication			
ACE inhibitor	0.99	0.16–6.24	0.9919
ARB	0.45	0.19–1.05	0.0654
ACE inhibitor, ARB, or both	0.46	0.20–1.08	0.0762
Beta-blocker	0.39	0.08–2.00	0.2601
Calcium channel blocker	0.38	0.14–1.01	0.0531
Diuretic	N.A		
Polycystic kidney disease characteristic			
ADPKD risk classification^a^	0.63	0.38–1.03	0.0663
Systolic blood pressure (mm Hg)	0.98	0.95–1.01	0.1622
Diastolic blood pressure (mm Hg)	0.99	0.95–1.02	0.3975
Mean blood pressure (mm Hg)	0.98	0.95–1.02	0.2665
TKV (mL)	1.00	1.00–1.00	0.5003
Height-adjusted TKV (mL/m)	1.00	1.00–1.00	0.7230
Urine albumin-to-creatinine ratio	0.97	0.93–1.02	0.2589
Kidney function			
Cr (mg/dL)	0.44	0.11–1.78	0.2514
CysC (mg/L)	0.31	0.04–2.73	0.2947
Urine osmolality (mOsm/kg)	1.00	1.00–1.00	0.4285
1/Cr [(mg/mL)^−1^]	1.01	1.00–1.02	0.1897
Ccr (mL/min)^b^	0.98	0.96–1.00	0.0503
eGFR CKD-EPI (mL/min/1.73 m^2^)^c^	1.00	0.97–1.02	0.8468
eGFR-J (mL/min/1.73 m^2^)^d^	0.99	0.97–1.02	0.6873

Factors were analyzed by logistic regression

*1/Cr* reciprocal of serum creatinine, *ACE* angiotensin-converting enzyme, *ADPKD* autosomal dominant polycystic kidney disease, *ARB* angiotensin II receptor blocker, *BMI* body mass index, *Ccr* estimated creatinine clearance, *CI* confidence interval, *CKD-EPI* Chronic Kidney Disease Epidemiology Collaboration, *Cr* serum creatinine, *CysC* serum cystatin C, *eGFR* estimated glomerular filtration rate, *eGFR-J* estimated glomerular filtration rate by the Japanese equation based on serum creatinine, *N.A.* not available, *TKV* total kidney volume

^a^ADPKD risk classification was defined on an ordinal scale from class 1B to 1E

^b^Ccr calculated by the Cockcroft–Gault formula

^c^eGFR calculated by the CKD-EPI equation modified for Japanese

^d^eGFR calculated by the Japanese eGFR equation based on Cr, as developed by the Japanese Society of Nephrology

**Table 3:** Multivariate analysis of factors predictive of TKV reduction

Factor	Odds ratio	95% CI	*P* value
Sex (male)	0.31	0.12–0.81	0.0165
BMI (kg/m^2^)	0.92	0.78–1.08	0.2984

Factors were analyzed by logistic regression

*BMI* body mass index, *CI* confidence interval, *TKV* total kidney volume

### Change in kidney function

Changes in eGFR (mean ± standard deviation [SD]) from baseline to year 3 in the placebo, responder, and non-responder groups were − 14.55 ± 8.26, − 9.13 ± 5.77, and − 9.31 ± 9.52 mL/min/1.73 m^2^, respectively, by the CKD-EPI equation and − 12.61 ± 7.23, − 8.47 ± 5.03, and − 8.58 ± 8.93 mL/min/1.73 m^2^, respectively, by the eGFR-J equation (Table 4).

**Table 4:** Changes in kidney function from baseline to year 3

Kidney function parameter	Group			*P* value		
	Placebo (*n* = 55)	Responders (*n* = 37)	Non-responders (*n* = 54)	PL vs R	PL vs NR	R vs NR
Cr (mg/dL)	0.28 ± 0.38	0.15 ± 0.16	0.17 ± 0.27	0.0853	0.1221	0.9311
CysC (mg/L)	0.20 ± 0.23	0.10 ± 0.11	0.14 ± 0.17	0.0297	0.2236	0.5334
1/Cr [(mg/mL)^−1^]	− 17.90 ± 12.57	− 12.12 ± 8.84	− 11.47 ± 14.35	0.0785	0.0217	0.9674
Ccr (mL/min)^a^	− 16.67 ± 11.31	− 10.81 ± 9.23	− 10.65 ± 13.73	0.0548	0.0237	0.9978
eGFR CKD-EPI (mL/min/1.73 m^2^)^b^	− 14.55 ± 8.26	− 9.13 ± 5.77	− 9.31 ± 9.52	0.0066	0.0032	0.9943
eGFR-J (mL/min/1.73 m^2^)^c^	− 12.61 ± 7.23	− 8.47 ± 5.03	− 8.58 ± 8.93	0.0269	0.0152	0.9970

Data are mean ± standard deviation. *P* values based on Tukey–Kramer’s honestly significant difference test are shown. Responders: tolvaptan-treated patients with a net decrease in TKV from baseline to year 3. Non-responders: tolvaptan-treated patients with a net increase in TKV from baseline to year 3

*1/Cr* reciprocal of serum creatinine, *Ccr* estimated creatinine clearance, *CKD-EPI* Chronic Kidney Disease Epidemiology Collaboration, *Cr* serum creatinine, *CysC* serum cystatin C, *eGFR* estimated glomerular filtration rate, *eGFR-J* estimated glomerular filtration rate by the Japanese equation based on serum creatinine, *NR* non-responder, *PL* placebo, *R* responder, *TKV* total kidney volume

^a^Ccr calculated by the Cockcroft–Gault formula

^b^eGFR calculated by the CKD-EPI equation modified for Japanese

^c^eGFR calculated by the Japanese eGFR equation based on Cr, as developed by the Japanese Society of Nephrology

**Fig. 5:**
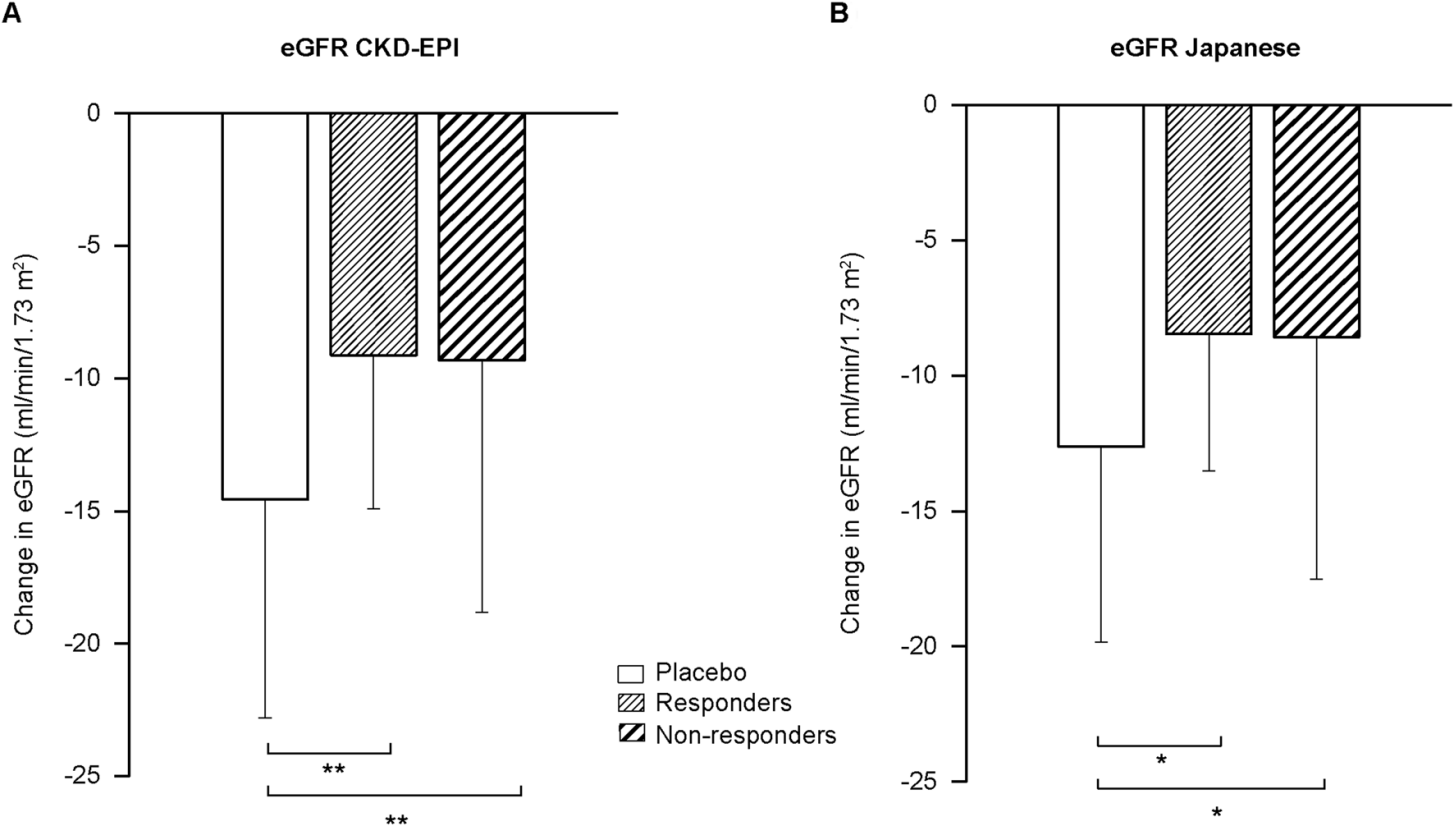
Change in eGFR from baseline to year 3. Values are expressed as mean and standard deviation. Responders: tolvaptan-treated patients with a net decrease in TKV from baseline to year 3. Non-responders: tolvaptan-treated patients with a net increase in TKV from baseline to year 3. *P < 0.05 and **P < 0.01 based on Tukey–Kramer’s honestly significant difference test comparing change in eGFR from baseline to year 3 between the placebo and responder groups or between the placebo and non-responder groups. *CKD-EPI* Chronic Kidney Disease Epidemiology Collaboration, *eGFR* estimated glomerular filtration rate, *eGFR Japanese* estimated glomerular filtration rate by the Japanese equation based on serum creatinine, *TKV* total kidney volume

Compared with the placebo group, the decline in eGFR assessed by the CKD-EPI (*P* = 0.0066, *P* = 0.0032) and eGFR-J (*P* = 0.0269, *P* = 0.0152) equations was significantly suppressed in both the responder and non-responder groups (Fig. 5). No statistically significant difference between the responder and non-responder groups was observed for any biomarker of renal function (eGFR CKD-EPI, eGFR-J, serum creatinine [Cr], reciprocal of serum creatinine [1/Cr], cystatin C [CysC], and Ccr) (Table 4). Changes in Ccr (mean ± SD) from baseline to year 3 in the placebo, responder, and non-responder groups were − 16.67 ± 11.31, − 10.81 ± 9.23, and − 10.65 ± 13.73 mL/min, respectively.

Data from additional analyses for changes in eGFR in class 1 patients and by age are summarized in Online Resources 1 and 2.

### Associations between TKV and renal function

In the placebo group, changes in Cr (*r* = 0.5971; *P* < 0.0001) and CysC (*r* = 0.5240; *P* < 0.0001) from baseline to year 3 demonstrated a significant positive correlation with TKV, while Ccr (*r* = − 0.3110; *P* = 0.0208) and eGFR CKD-EPI (*r* = − 0.2811; *P* = 0.0376) showed a significant negative correlation (Table 5). However, no statistically significant associations between changes in TKV and renal function were observed in either the responder or the non-responder group.

**Table 5:** Correlations between changes in TKV and kidney function

Group	Kidney function parameter	*n*	Univariate		Correlation coefficient^a^	Regression analysis			*P* value
			Mean	SD	*r*	Estimate	SE	95% CI	
Placebo	Cr (mg/dL)	55	0.28	0.38	0.5971	28.02	5.17	17.65 to 38.39	< 0.0001
	CysC (mg/L)	55	0.20	0.23	0.5240	40.72	9.09	22.49 to 58.96	< 0.0001
	1/Cr [(mg/mL)^−1^]	55	− 17.90	12.57	− 0.1518	− 0.21	0.19	− 0.60 to 0.17	0.2687
	Ccr (mL/min)^b^	55	− 16.67	11.31	− 0.3110	− 0.48	0.20	− 0.89 to − 0.08	0.0208
	eGFR CKD-EPI (mL/min/1.73 m^2^)^c^	55	− 14.55	8.26	− 0.2811	− 0.60	0.28	− 1.17 to − 0.04	0.0376
	eGFR-J (mL/min/1.73 m^2^)^d^	55	− 12.61	7.23	− 0.2333	− 0.57	0.33	− 1.22 to 0.08	0.0865
Responders	Cr (mg/dL)	37	0.15	0.16	0.3232	10.48	5.18	− 0.05 to 21.00	0.0510
	CysC (mg/L)	37	0.10	0.11	0.1922	9.13	7.88	− 6.86 to 25.11	0.2544
	1/Cr [(mg/mL)^−1^]	37	− 12.12	8.84	− 0.1750	− 0.10	0.10	− 0.29 to 0.09	0.3001
	Ccr (mL/min)^b^	37	− 10.81	9.23	− 0.2161	− 0.12	0.09	− 0.30 to 0.07	0.1988
	eGFR CKD-EPI (mL/min/1.73 m^2^)^c^	37	− 9.13	5.77	− 0.2967	− 0.26	0.14	− 0.55 to 0.03	0.0746
	eGFR-J (mL/min/1.73 m^2^)^d^	37	− 8.47	5.03	− 0.1661	− 0.17	0.17	− 0.51 to 0.17	0.3258
Non-responders	Cr (mg/dL)	54	0.17	0.27	0.1856	8.28	6.08	− 3.92 to 20.48	0.1791
	CysC (mg/L)	54	0.14	0.17	0.1764	12.46	9.64	− 6.88 to 31.81	0.2019
	1/Cr [(mg/mL)^−1^]	54	− 11.47	14.35	− 0.1788	− 0.15	0.11	− 0.37 to 0.08	0.1959
	Ccr (mL/min)^b^	54	− 10.65	13.73	− 0.1139	− 0.10	0.12	− 0.34 to 0.14	0.4124
	eGFR CKD-EPI (mL/min/1.73 m^2^)^c^	54	− 9.31	9.52	− 0.2302	− 0.29	0.17	− 0.62 to 0.05	0.0940
	eGFR-J (mL/min/1.73 m^2^)^d^	54	− 8.58	8.93	− 0.2043	− 0.27	0.18	− 0.63 to 0.09	0.1384

Mean and SD represent changes in kidney function parameters from baseline to year 3. Correlation coefficient, estimate, SE, 95% CI, and *P* value show correlations between changes in TKV and kidney function parameters from baseline to year 3. Responders: tolvaptan-treated patients with a net decrease in TKV from baseline to year 3. Non-responders: tolvaptan-treated patients with a net increase in TKV from baseline to year 3

*1/Cr* reciprocal of serum creatinine, *Ccr* estimated creatinine clearance, *CI* confidence interval, *CKD-EPI* Chronic Kidney Disease Epidemiology Collaboration, *Cr* serum creatinine, *CysC* serum cystatin C, *eGFR* estimated glomerular filtration rate, *eGFR-J* estimated glomerular filtration rate by the Japanese equation based on serum creatinine, *SD* standard deviation, *SE* standard error, *TKV* total kidney volume

^a^Pearson's correlation coefficient

^b^Ccr calculated by the Cockcroft–Gault formula

^c^eGFR calculated by the CKD-EPI equation modified for Japanese

^d^eGFR calculated by the Japanese eGFR equation based on Cr, as developed by the Japanese Society of Nephrology

### Urine osmolality

We examined changes in urine osmolality during the trial. Mean ± SD urine osmolalities in the placebo, responder, and non-responder groups were 475.0 ± 137.1, 440.8 ± 140.6, and 465.7 ± 151.2 mOsm/kg, respectively, at baseline and 413.4 ± 132.5, 250.4 ± 114.6, and 247.7 ± 137.1 mOsm/kg, respectively, at month 36. Mean urine osmolality decreased significantly more in the tolvaptan-treated groups compared with the placebo group, but there were no differences between the responder group and the non-responder group at 12, 24, and 36 months (Table 6).

**Table 6:** Changes in urine osmolality from baseline to year 3

Urine osmolality (mOsm/kg)	Group			*P* value		
	Placebo	Responders	Non-responders	PL vs R	PL vs NR	R vs NR
Baseline	475.0 ± 137.1 (*n* = 53)	440.8 ± 140.6 (*n* = 36)	465.7 ± 151.2 (*n* = 55)	0.5149	0.9398	0.6987
Month 12	401.2 ± 160.3 (*n* = 53)	214.7 ± 99.5 (*n* = 36)	249.7 ± 140.9 (*n* = 52)	< 0.0001	< 0.0001	0.4830
Month 24	420.1 ± 160.0 (*n* = 53)	239.4 ± 103.6 (*n* = 34)	234.8 ± 108.3 (*n* = 53)	< 0.0001	< 0.0001	0.9854
Month 36	413.4 ± 132.5 (*n* = 53)	250.4 ± 114.6 (*n* = 36)	247.7 ± 137.1 (*n* = 55)	< 0.0001	< 0.0001	0.9949

Data are mean ± standard deviation. *P* values based on Tukey–Kramer’s honestly significant difference test are shown. Responders: tolvaptan-treated patients with a net decrease in TKV from baseline to year 3. Non-responders: tolvaptan-treated patients with a net increase in TKV from baseline to year 3

*NR* non-responder, *PL* placebo, *R* responder, *TKV* total kidney volume

## Discussion

Mechanisms of cyst expansion in ADPKD and the associated increase in TKV are based on reduced intracellular Ca^2+^ influx caused by mutations in either *PKD1* or *PKD2,* increased cellular adenosine 3′, 5′-cyclic monophosphate (cAMP) levels, and aberrant Ras/Raf/ERK activation [18]. Tolvaptan blocks the vasopressin V_2_-receptor, reducing cAMP levels [18]. The progression of ADPKD is characterized by a continuous expansion of cyst volume, a process that was well represented by change in TKV over time in the placebo group of this study (Fig. 3). During longitudinal follow-up in CRISP II, htTKV and GFR exhibited a significant negative correlation [4]. This phenomenon has also been observed in a Japanese cohort, in which a significant negative correlation between baseline TKV and eGFR slope was observed [19]. Thus, TKV is used as a surrogate biomarker of ADPKD progression [1].

In our study, female sex was a significant positive predictor of tolvaptan’s effect in reducing TKV growth. Since there was no modification of tolvaptan dosage for either sex in this study, it is possible that the same dosage may have produced higher serum drug concentrations in females. However, pharmacokinetic analyses in patients with ADPKD have shown that there are no sex differences in the blood concentration of tolvaptan [20]; therefore, we could not define why female sex was identified as a predictor.

In a previous post hoc analysis of TEMPO 3:4, it was reported that baseline urine osmolality influenced disease progression and the response to tolvaptan in patients with ADPKD [21]. The importance of vasopressin is further supported by recent findings on copeptin levels, a surrogate marker for arginine vasopressin (AVP). Copeptin concentrations are higher in men, positively correlate with TKV growth, and predict response to tolvaptan in ADPKD [22, 23]. Subsequent research has confirmed that men have a consistently higher urine osmolality than women [24]. In the present analysis, however, urine osmolality did not predict the effect of tolvaptan in reducing TKV growth. Additionally, there were no differences in urine osmolality at years 1, 2, and 3 between the responder and non-responder groups.

ADPKD risk classification is a practical method for evaluating the prognosis of patients with ADPKD [12, 25, 26]. In the model developed by Irazabal and colleagues, rates of TKV growth and eGFR decline increase stepwise from class 1A to 1E in ADPKD with a typical presentation. We assessed if there was any difference in risk class distribution between Japanese patients and the global population. In our analysis, classes 1B, 1C, 1D, and 1E constituted 9.5%, 40.8%, 30.6%, and 16.3%, respectively, of the total 147 patients (Table 1), which is a similar distribution to that in the TEMPO 3:4 global population (7.6%, 38.2%, 35.4%, 18.8% for classes 1B, 1C, 1D, and 1E, respectively) [25]. Taken together, our findings indicate no reason to suggest that the effects of tolvaptan in slowing renal function decline are dependent on changes in TKV growth rate. However, the non-responder group included more class 1E patients compared with the responder group (8.1% vs 23.6%). We cannot deny the possibility that TKV growth in the non-responder group might have been much faster and masked the efficacy of tolvaptan.

Several lines of evidence suggest that vasopressin contributes to non-diabetic and diabetic chronic kidney disease progression. Plasma vasopressin levels are increased in animal models and in patients with non-diabetic chronic kidney disease, in animal models of streptozotocin-induced and genetic diabetes mellitus, and in patients with type I and type II diabetes mellitus [27, 28]. Plasma levels of copeptin are inversely correlated with GFR [29]. Previous studies have shown that AVP is associated with the progression of chronic kidney disease. Plasma copeptin levels are associated with a decline in kidney function in recipients of kidney transplant [30], and high plasma copeptin levels are associated with lower GFR in patients with type II diabetes mellitus [31]. Furthermore, treatment with AVP V_1a_ and V_2_ receptor antagonists has been shown to cause a significant reduction in blood pressure, proteinuria, and glomerulosclerosis in animal models with 5/6 nephrectomy [32]. Although the underlying mechanisms are unknown, inhibiting the AVP V_2_-receptor may confer a renal protective effect, independent of the kidney volume reduction. Thus, tolvaptan might plausibly be beneficial for patients with ADPKD in whom there is little treatment effect on TKV.

Limitations of this study are its small size and use of a sample from a single ethnic group. Potential differences in genetic features between responders and non-responders warrant further investigation.

Currently, in Japan and other countries, the labeling for tolvaptan in ADPKD stresses its effect on reducing the rate of TKV increase. In the United States, following the Replicating Evidence of Preserved Renal Function: an Investigation of Tolvaptan Safety and Efficacy in ADPKD (REPRISE) trial [33], which did not assess changes in TKV, the Food and Drug Administration approved tolvaptan with an indication to slow renal function decline. In our analysis of the Japanese cohort of TEMPO 3:4, we have shown that tolvaptan suppressed eGFR decline regardless of the effects on TKV growth. Accordingly, we propose that patients receiving treatment with tolvaptan who do not experience a reduction in the rate of TKV growth should not discontinue treatment as there may still be beneficial effects on slowing of renal function decline.

## Supplementary Information

Below is the link to the electronic supplementary material.

Supplementary file1 (PDF 186 KB)
