# Supplementary material for: Preservation of kidney function irrelevant of total kidney volume growth rate with tolvaptan treatment in patients with autosomal dominant polycystic kidney disease
Source: Clin Exp Nephrol. 2021 Jan 20;25(5):467–78. doi: 10.1007/s10157-020-02009-0 (PMC8038960; doi:10.1007/s10157-020-02009-0)
Supplement: Supplementary file 1 — Supplementary file1 (PDF 186 KB) [file 10157_2020_2009_MOESM1_ESM.pdf]

Electronic Supplementary Material

Journal: ***Clinical and Experimental Nephrology***

Preservation of kidney function irrelevant of total kidney volume growth rate with tolvaptan treatment in patients with autosomal dominant polycystic kidney disease

Shigeo Horie[1,2], Satoru Muto[1,2], Haruna Kawano[1,2], Tadashi Okada[3], Yoshiyuki Shibasaki[4], Koji Nakajima[4], Tatsuki Ibuki[4]

[1]Department of Advanced Informatics for Genetic Diseases, Juntendo University Graduate School of Medicine, Tokyo, Japan; [2]Department of Urology, Juntendo University Graduate School of Medicine, Tokyo, Japan; [3]Department of Clinical Development, Otsuka Pharmaceutical Co., Ltd., Osaka, Japan; [4]Medical Affairs, Otsuka Pharmaceutical Co., Ltd., Tokyo, Japan

Correspondence:

Shigeo Horie, MD, PhD

Department of Urology, Juntendo University Graduate School of Medicine

2-1-1 Hongo, Bunkyo-ku, Tokyo 113-8421, Japan

E-mail: shorie@juntendo.ac.jp

Telephone: +81-3-5802-1227

Fax: +81-3-5803-1227

Online Resource 1 Change in eGFR from baseline to year 3 in class 1 patients

	Group			*P* value		
Class	Placebo	Responders	Non-responders	PL vs R	PL vs NR	R vs NR
		eGFR CKD-EPI (mL/min/1.73 m^2)[a]				
1A	-	-	-	-	-	-
1B	-6.22 ± 7.26 (n = 4)	-10.30 ± 6.90 (n = 6)	-2.65 ± 7.69 (n = 3)	0.6633	0.7957	0.3279
1C	-13.82 ± 7.53 (n = 26)	-8.21 ± 5.99 (n = 13)	-8.03 ± 11.40 (n = 21)	0.1566	0.0737	0.9981
1D	-17.71 ± 8.77 (n = 17)	-10.07 ± 5.89 (n = 11)	-9.17 ± 7.47 (n = 17)	0.0357	0.0064	0.9511
1E	-14.40 ± 7.73 (n = 8)	-12.37 ± 5.20 (n = 3)	-13.10 ± 8.37 (n = 13)	0.9246	0.9301	0.9886
		eGFR-J (mL/min/1.73 m^2)[b]				
1A	-	-	-	-	-	-
1B	-6.33 ± 6.74 (n = 4)	-9.22 ± 7.24 (n = 6)	-4.89 ± 9.28 (n = 3)	0.8277	0.9665	0.7061
1C	-11.25 ± 5.92 (n = 26)	-7.68 ± 5.22 (n = 13)	-7.54 ± 9.72 (n = 21)	0.3339	0.2068	0.9983
1D	-16.24 ± 8.22 (n = 17)	-9.66 ± 4.50 (n = 11)	-7.39 ± 8.05 (n = 17)	0.0689	0.0034	0.7120
1E	-12.44 ± 6.52 (n = 8)	-10.26 ± 4.36 (n = 3)	-12.69 ± 8.26 (n = 13)	0.9023	0.9969	0.8674

Data are mean ± standard deviation. *P* values based on Tukey-Kramer’s honestly significant difference test are shown.

Responders: tolvaptan-treated patients with a net decrease in TKV from baseline to year 3.

Non-responders: tolvaptan-treated patients with a net increase in TKV from baseline to year 3.

[a]eGFR calculated by the CKD-EPI equation modified for Japan.

[b]eGFR calculated by the Japanese eGFR equation based on serum creatinine, as developed by the Japanese Society of Nephrology.

CKD-EPI, Chronic Kidney Disease Epidemiology Collaboration; eGFR, estimated glomerular filtration rate; eGFR-J, estimated glomerular filtration rate by the Japanese equation based on serum creatinine; NR, non-responder; PL, placebo; R, responder; TKV, total kidney volume.

Online Resource 2 Change in eGFR from baseline to year 3 by age

	Group			*P* value		
Age (years)	Placebo	Responders	Non-responders	PL vs R	PL vs NR	R vs NR
		eGFR CKD-EPI (mL/min/1.73 m^2)[a]				
≥ 20 to < 30	-3.01 ± 1.08 (n = 2)	-18.37 (n = 1)	-7.62 ± 6.48 (n = 7)	0.1624	0.6246	0.2802
≥ 30 to < 40	-14.26 ± 7.02 (n = 22)	-10.64 ± 5.38 (n = 19)	-8.79 ± 11.73 (n = 23)	0.3820	0.0951	0.7718
≥ 40 to < 50	-15.51 ± 8.85 (n = 31)	-6.56 ± 5.36 (n = 16)	-10.30 ± 8.02 (n = 24)	0.0014	0.0474	0.3142
≥ 50 to < 60	-	-12.44 (n = 1)	-	-	-	-
		eGFR-J (mL/min/1.73 m^2)[b]				
≥ 20 to < 30	-6.49 ± 4.16 (n = 2)	-15.30 (n = 1)	-7.86 ± 6.34 (n = 7)	0.4992	0.9577	0.5198
≥ 30 to < 40	-12.01 ± 5.74 (n = 22)	-10.78 ± 4.21 (n = 19)	-8.80 ± 10.68 (n = 23)	0.8617	0.3374	0.6801
≥ 40 to < 50	-13.42 ± 8.18 (n = 31)	-5.22 ± 4.26 (n = 16)	-8.59 ± 8.00 (n = 24)	0.0018	0.0502	0.3432
≥ 50 to < 60	-	-9.78 (n = 1)	-	-	-	-

Data are mean ± standard deviation. *P* values based on Tukey-Kramer’s honestly significant difference test are shown.

Responders: tolvaptan-treated patients with a net decrease in TKV from baseline to year 3.

Non-responders: tolvaptan-treated patients with a net increase in TKV from baseline to year 3.

[a]eGFR calculated by the CKD-EPI equation modified for Japan.

[b]eGFR calculated by the Japanese eGFR equation based on serum creatinine, as developed by the Japanese Society of Nephrology.

CKD-EPI, Chronic Kidney Disease Epidemiology Collaboration; eGFR, estimated glomerular filtration rate; eGFR-J, estimated glomerular filtration rate by the Japanese equation based on serum creatinine; NR, non-responder; PL, placebo; R, responder; TKV, total kidney volume.
